# Supplementary material for: The metastatic capacity of high-grade serous ovarian cancer cells changes along disease progression: inhibition by mifepristone
Source: Cancer Cell Int. 2022 Dec 9;22:397. doi: 10.1186/s12935-022-02822-5 (PMC9733158; doi:10.1186/s12935-022-02822-5)
Supplement: Supplementary file 1 — Additional file 1: Table S1. Source and dilutions of antibodies utilized in this work. [file 12935_2022_2822_MOESM1_ESM.docx]

**Table S1.** Source and dilutions of antibodies utilized in this work.

| **Antibody** | **Company** | **Catalogue** | **Host** | **Concentration** | |
| --- | --- | --- | --- | --- | --- |
|  |  |  |  | **IF** | **WB** |
| Phospho-Histone H3 | EMD Millipore Corporation | 06-570 | Rabbit | 1:50 | NA |
| E-Cadherin | Cell Signaling Technology | 24E10 | Rabbit | 1:50 | 1:1000 |
| N-Cadherin | Cell Signaling Technology | 13A9 | Mouse | 1:50 | 1:1000 |
| CD44 | Novocastra Laboratories | NCL-CD44 | Mouse | 1:100 | NA |
| CD44 | Spring Bioscience | E17370 | Rabbit | NA | 1:1000 |
| Calretinin | EMD Millipore Corporation | MAB1568 | Mouse | 1:100 | NA |
| Vimentin | Cell Signaling Technology | 5741S | Rabbit | 1:100 | 1:1000 |
| Cytokeratin 7 | Novus Biologicals | NBP1-33104 | Rabbit | NA | 1:1000 |
| EpCAM | Cell Signaling Technology | D9S3P | Rabbit | NA | 1:1000 |
| Glucocorticoid Receptor (E-20) | Santa Cruz Biotechnology | Sc-1003 | Rabbit | NA | 1:1000 |
| Progesterone Receptor (hPRa7) | Thermo Scientific | MS-197-P | Mouse | NA | 4 μg/ml |
| β-actin | Sigma-Aldrich | A5441-2ML | Mouse | NA | 1:10000 |
| Alexa Fluor 488 (anti-rabbit) | Invitrogen | A11034 | Goat | 1:1000 | NA |
| Alexa Fluor 488 (anti-mouse) | Invitrogen | A11001 | Goat | 1:1000 | NA |
| Rhodamine Red (anti-mouse) | ThermoFisher Scientific | 31660 | Goat | 1:1000 | NA |
| Alexa Fluor 594 (anti-rabbit) | Invitrogen | A32754 | Donkey | 1:1000 | NA |
| Anti-Rabbit HRP Conjugate | BioRad Laboratories Inc | 170-6515 | Goat | NA | 1:10000 |
| Anti-Mouse HRP Conjugate | BioRad Laboratories Inc | 170-6516 | Goat | NA | 1:8000 |

IF: immunofluorescence; WB: western blotting
