# Supplementary figures and images for: The metastatic capacity of high-grade serous ovarian cancer cells changes along disease progression: inhibition by mifepristone
Source: Cancer Cell Int. 2022 Dec 9;22:397. doi: 10.1186/s12935-022-02822-5 (PMC9733158; doi:10.1186/s12935-022-02822-5)

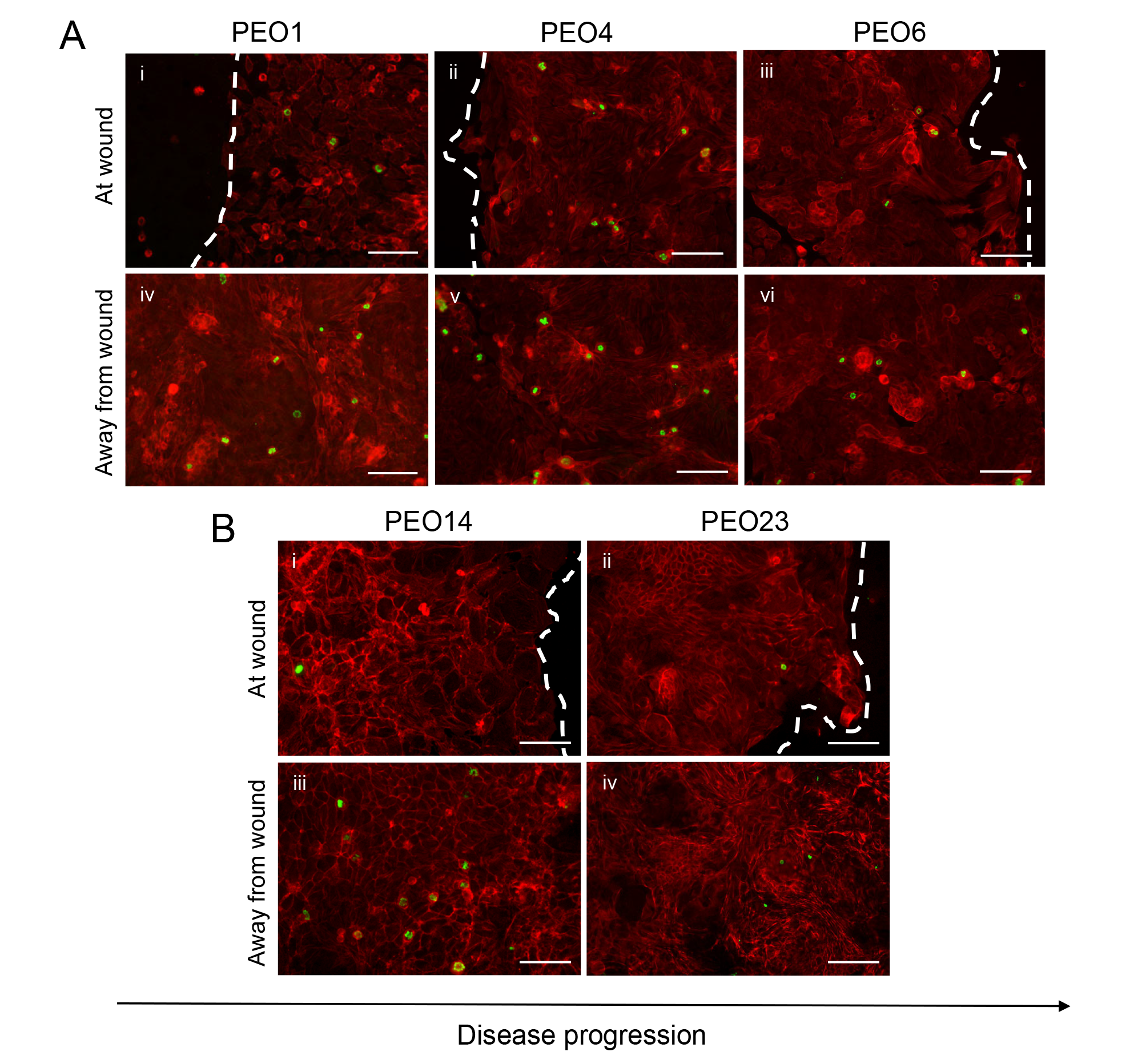

Supplement: Supplementary file 2 — Additional file 2: Fig. S1. Cells migrating into the wound express no phospho-histone H3 (pHH3) compared to an increase in positivity away from the wound, demonstrating that cells are migrating into the wound and not proliferating. Cells were fixed after 36 h and stained for pHH3 (green) by immunofluorescence, and Alexa Fluor-594 Phalloidin to stain for the cytoskeleton. The white dashed lines represent the front of the wound. Scale bar = 100 µm. [file 12935_2022_2822_MOESM2_ESM.tif]

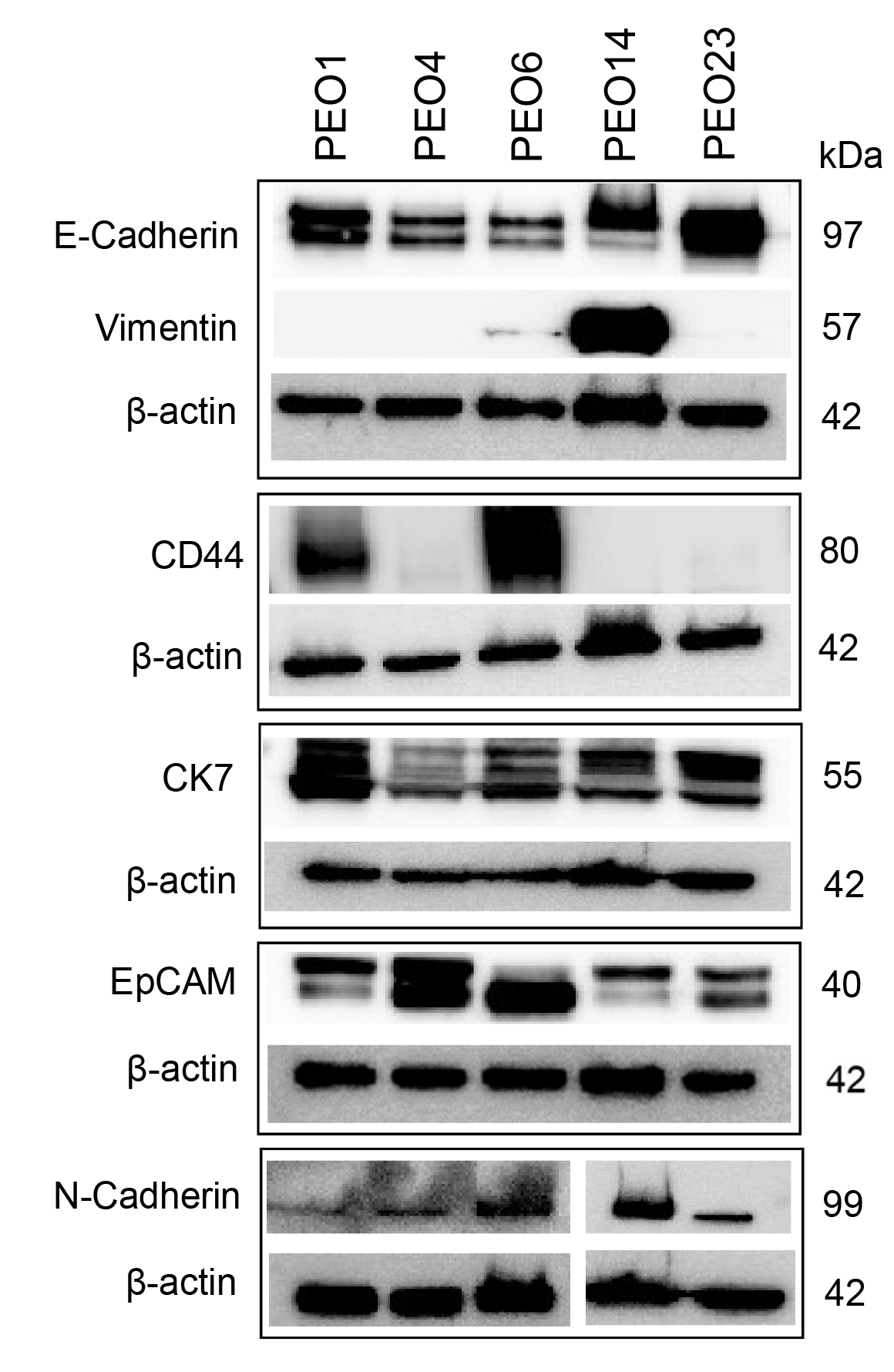

Supplement: Supplementary file 3 — Additional file 3: Fig. S2. Expression of EMT related molecules by western blotting in cells obtained from patient 1 (PEO1, PEO4 and PEO6) or patient 2 (PEO14 and PEO23). Results show positivity of expression of E-cadherin, CK7, EpCAM and N-cadherin in all cell lines studied; the expression of CD44 was only observed in PEO1 and PEO6 cells, whereas the expression of vimentin was only positive in PEO6 and PEO14 cells. [file 12935_2022_2822_MOESM3_ESM.tif]

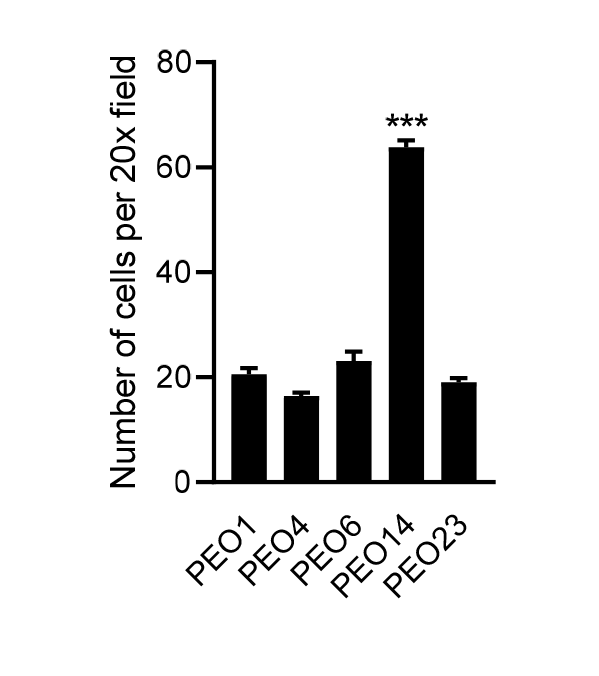

Supplement: Supplementary file 4 — Additional file 4: Fig. S3. The adhesion rate of HGSOC cells to fibronectin only changes along disease progression in one case. Plates were pre-coated with fibronectin and cells were left to adhere for 2 h. Data shown represents the mean ± s.e.m. Statistical analysis was done using one-way ANOVA followed by Bonferroni’s test. ***P < 0.001 compared to the other cell lines. [file 12935_2022_2822_MOESM4_ESM.tif]

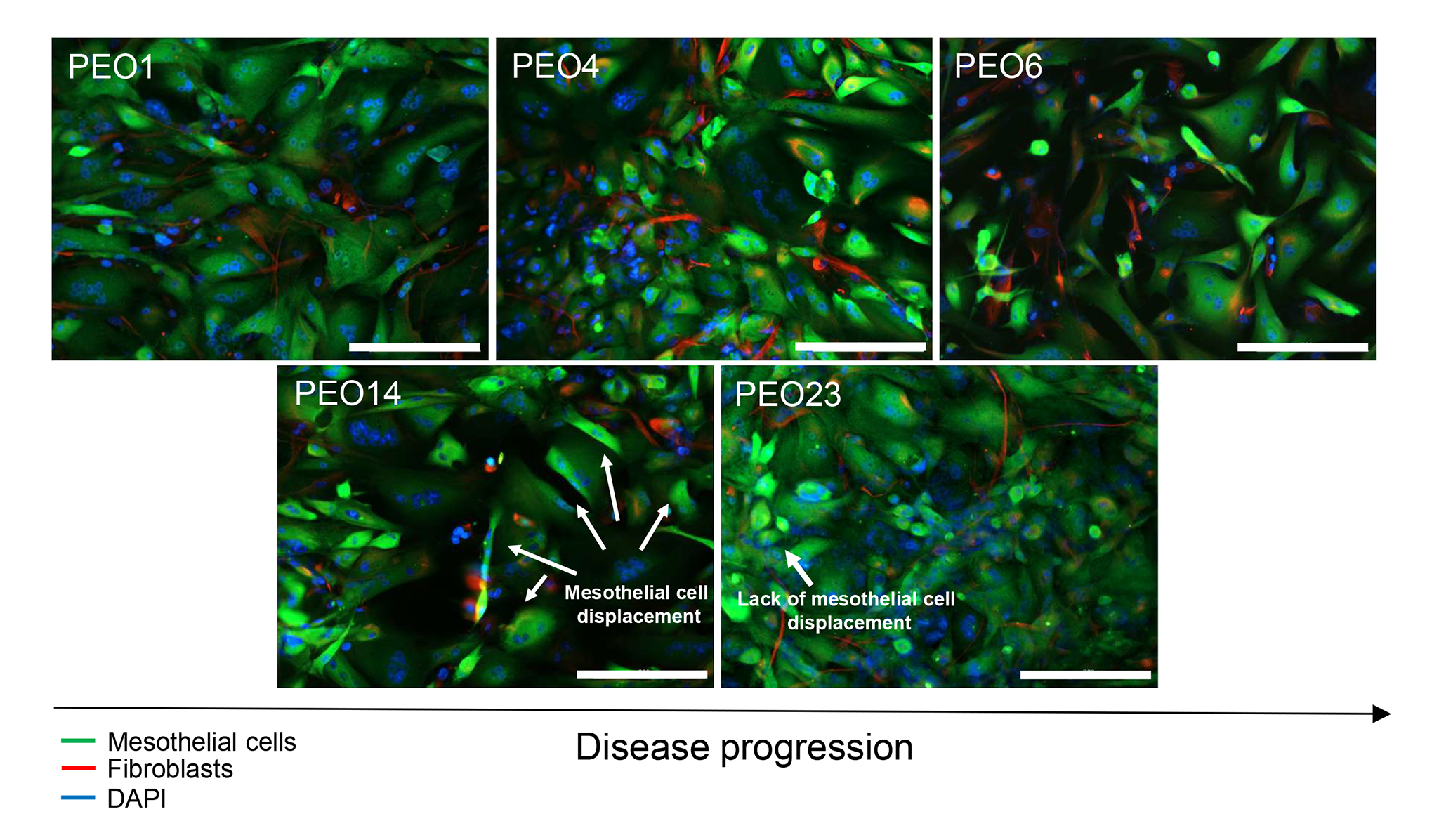

Supplement: Supplementary file 5 — Additional file 5: Fig. S4. The addition of conditioned media to organotypic models causes a displacement of the mesothelial cell monolayer in PEO4, PEO6, and PEO14. Panels are a visual representation of organotypic models incubated with conditioned media from the 5 HGSOC cell lines studied, for 24 h. Cells were fixed with 4% PFA and stained for calretinin (green, [mesothelial cells]), vimentin (red, [fibroblasts]) by immunofluorescence, and DAPI (blue), to stain the nuclei. Scale bar = 200 µm. In the PEO14 panel, arrows represent mesothelial cells that are displaced, whereas in the PEO23 panel, the short arrow represents a cluster of mesothelial cells that is not displaced. [file 12935_2022_2822_MOESM5_ESM.tif]

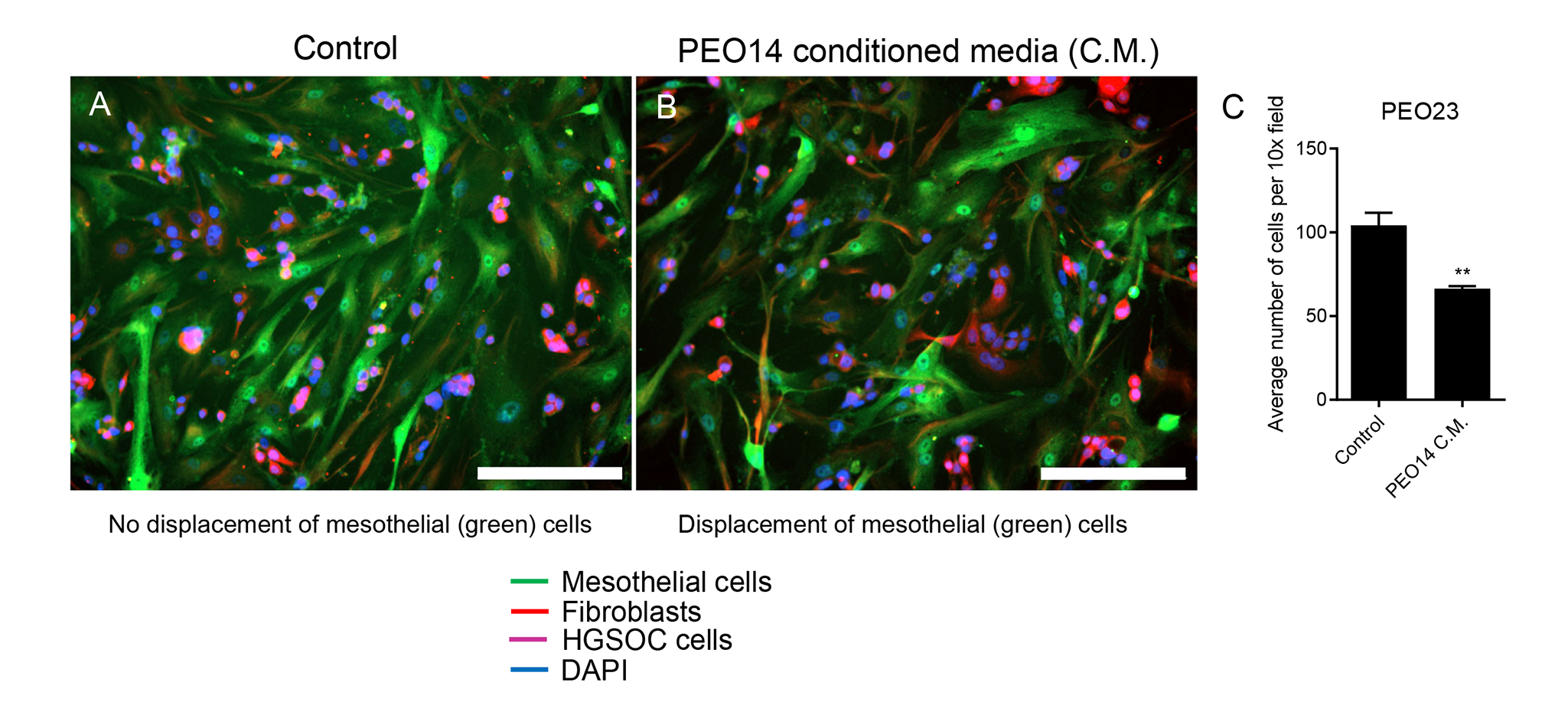

Supplement: Supplementary file 6 — Additional file 6: Fig. S5. The adhesion rate of PEO23 to an organotypic model, composed of fibroblasts embedded in collagen I topped with a monolayer of LP9 cells, decreases when incubated with PEO14 conditioned media. Mesothelial clearance is increased in the presence of PEO14 conditioned media (C.M.). Panels A and B are a visual representation of PEO23 adhered to the LP9 monolayer incubated without (A) or with (B) PEO14 C.M. for 24 h. PEO23 cells were incubated with Cell Tracker™ Deep Red before plating. Cells were fixed with 4% PFA and stained for calretinin (green, [mesothelial cell]s), vimentin (red, [fibroblast]s) by immunofluorescence, and DAPI (blue), to stain the nuclei. Scale bar = 200 µm. For panel (C) **P < 0.01 compared to control. Statistical analysis was done using student t-test. [file 12935_2022_2822_MOESM6_ESM.tif]

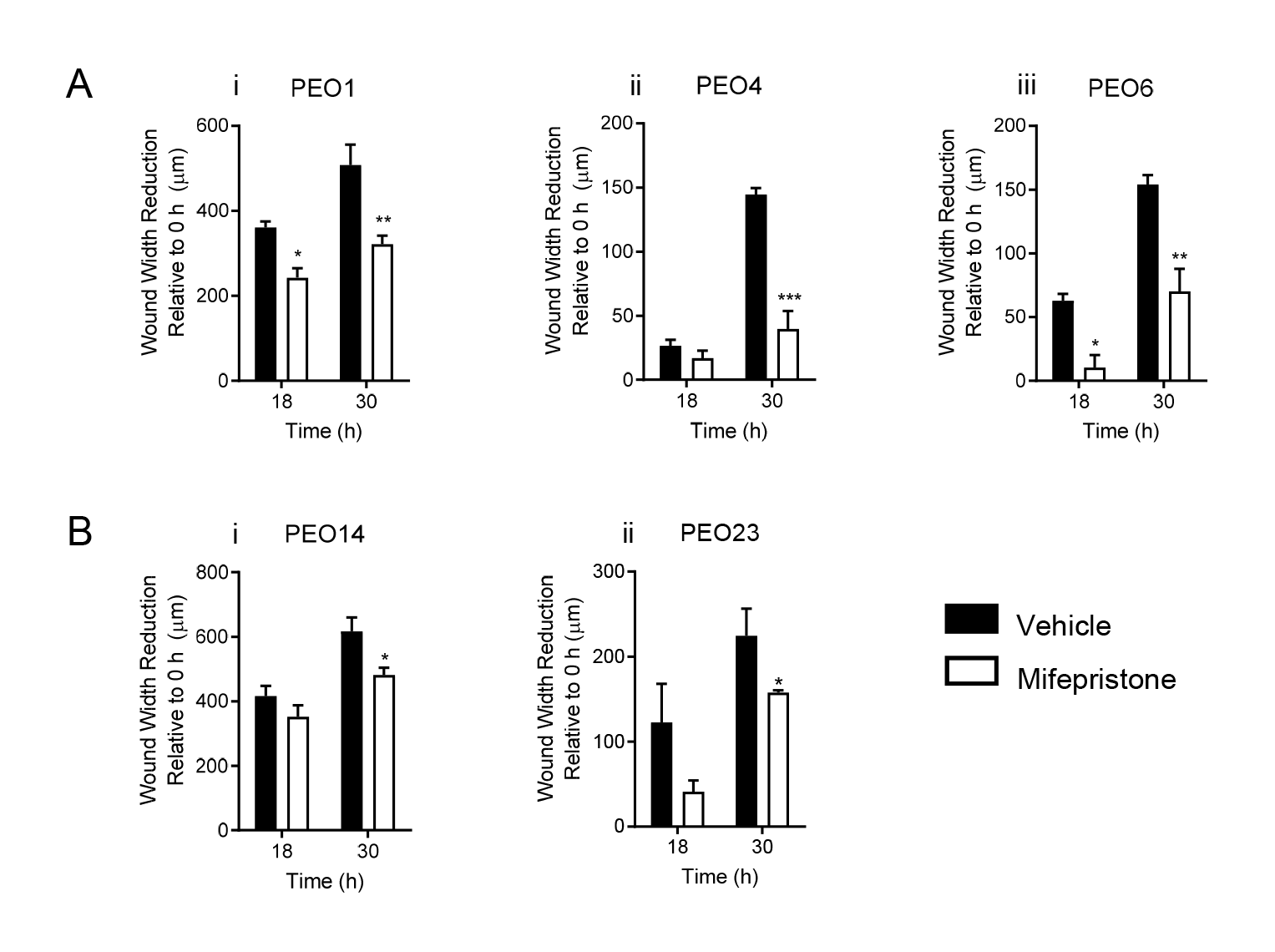

Supplement: Supplementary file 7 — Additional file 7: Fig. S6. Mifepristone inhibits the migration capacity of all five HGSOC in a wound healing assay, despite basal capabilities. PEO1, PEO4 and PEO6 series, panel (A[i-iii]) and PEO14 and PEO23 series, panel (B [i-ii]). HGSOC cells were treated with 20 µM of MF for 72 h prior to plating. Data shown represents the mean ± s.e.m. *P < 0.05, **P < 0.01, ***P < 0.001 compared to Vehicle. Vehicle (closed bars), MF (open bars). Statistical analysis was done using two-way ANOVA followed by Bonferroni’s test. [file 12935_2022_2822_MOESM7_ESM.tif]

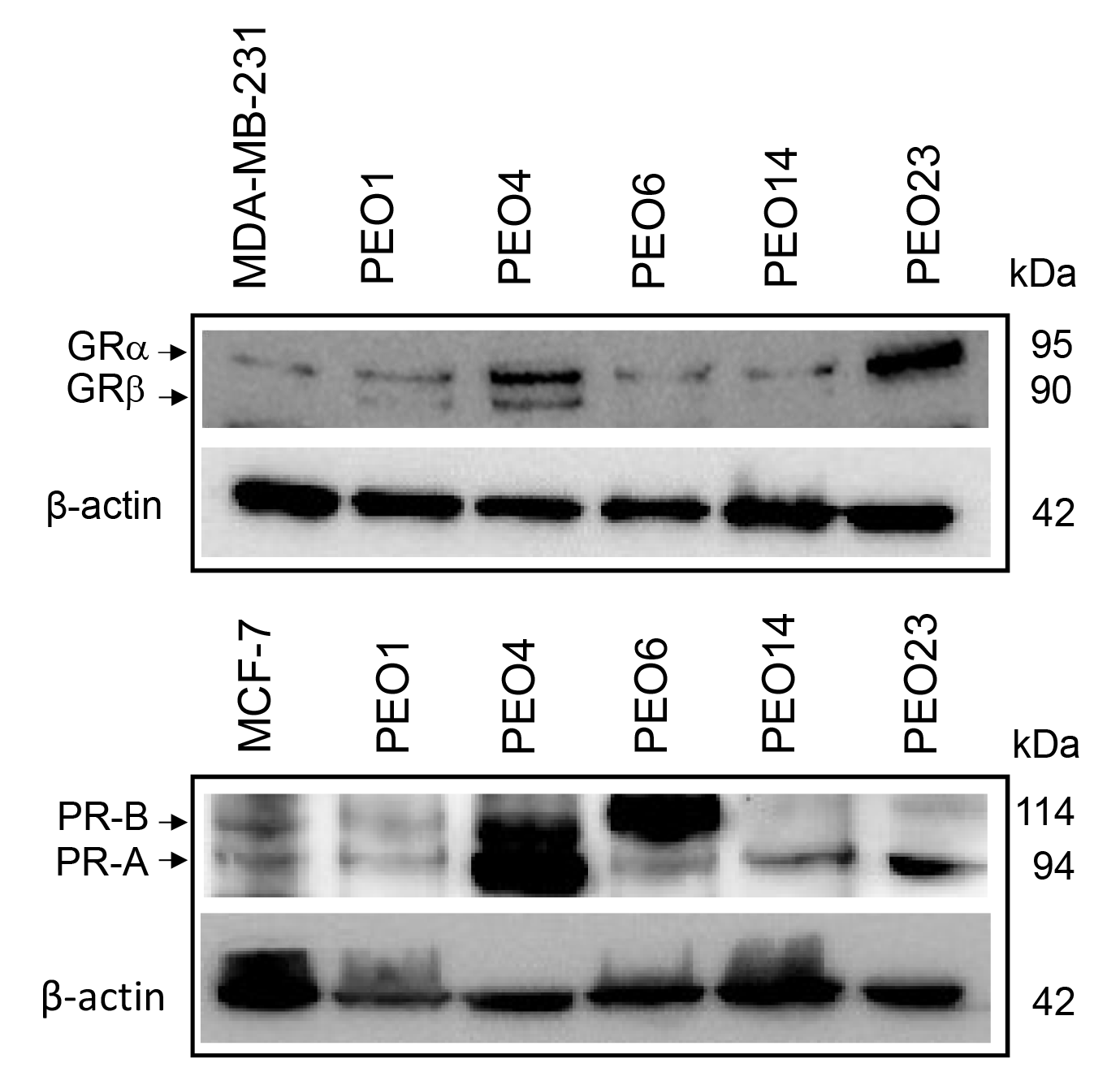

Supplement: Supplementary file 8 — Additional file 8: Fig. S7. Expression of glucocorticoid receptor (GR) isoforms alpha (95 kDa) and beta (90 kDa) (upper panel) and of progesterone receptor (PR) isoforms B (114 kDa) and A (94 kDa) (lower panel). We utilized the breast cancer cell line MDA-MB-231 as a positive control for GR whereas another breast cancer cell line, MCF-7, was used as a positive control for PR. GRα was apparent in all cell lines whereas GRβ was only visible in PEO1 and PEO4 cells. As for PR, results show expression of PR-A and PR-B in MCF-7, PEO1, PEO4 and PEO6, while PEO14 and PEO23 express mostly PR-A. [file 12935_2022_2822_MOESM8_ESM.tif]
