# Supplementary material for: The metastatic capacity of high-grade serous ovarian cancer cells changes along disease progression: inhibition by mifepristone
Source: Cancer Cell Int. 2022 Dec 9;22:397. doi: 10.1186/s12935-022-02822-5 (PMC9733158; doi:10.1186/s12935-022-02822-5)
Supplement: Supplementary file 9 — Additional file 9: Fig. S8. Uncropped raw western blot results in whole membranes pertaining to Additional file 3: Fig S2 and Additional file 8: Fig. S7. [file 12935_2022_2822_MOESM9_ESM.pdf]

Figure S2

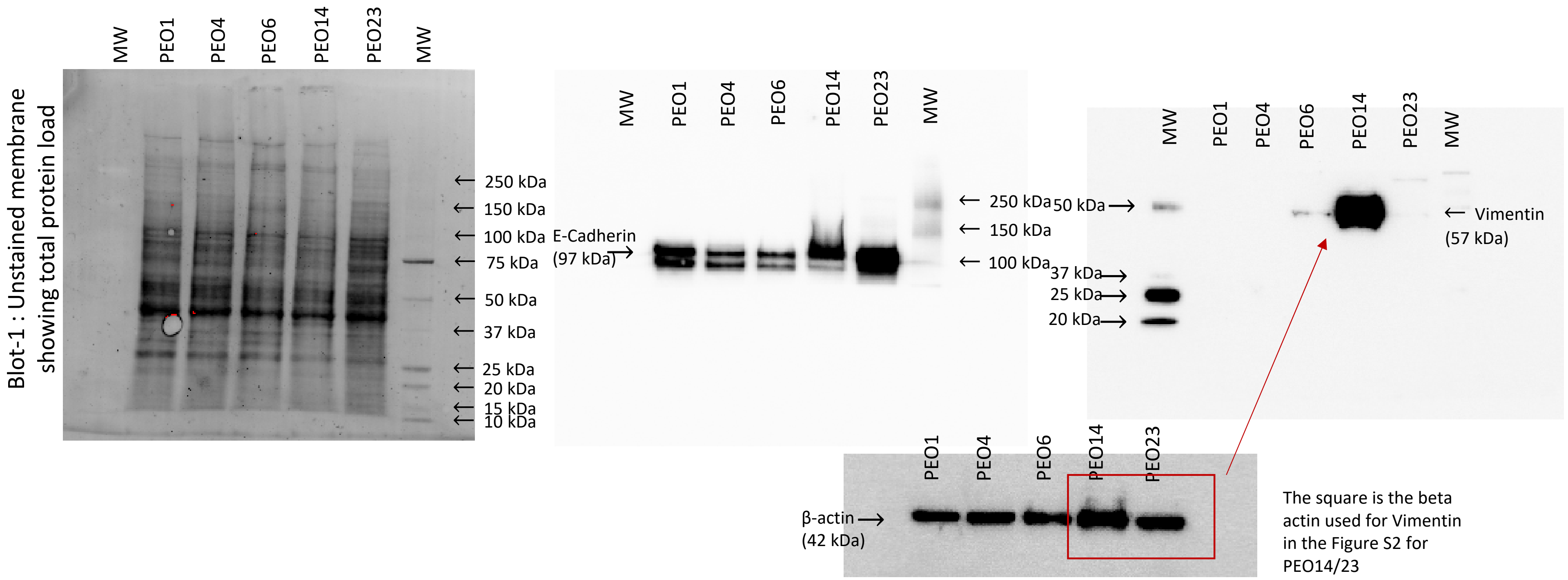

Proteins collected from HGSOC cells were resolved on a 10% gel (TGX stain free fast cast acrylamide) via electrophoresis (200 V). The gel was then activated using UV light and a Trans-Blot® Turbo™ Transfer System was used to transfer the resolved proteins to a PVDF membrane. After transfer, total protein load was visualized on the unstained membrane using BioRad ChemiDoc imager. After 1 hour of blocking with 5% non-fat dry milk, blot 1 was cut at75 kDa. The part below 75 kDa was incubated for vimentin and β-actin and the part above 75 kDa was incubated for E-Cadherin. Two stained molecular weight ladders on each side were used for cutting accuracy.

Figure S2

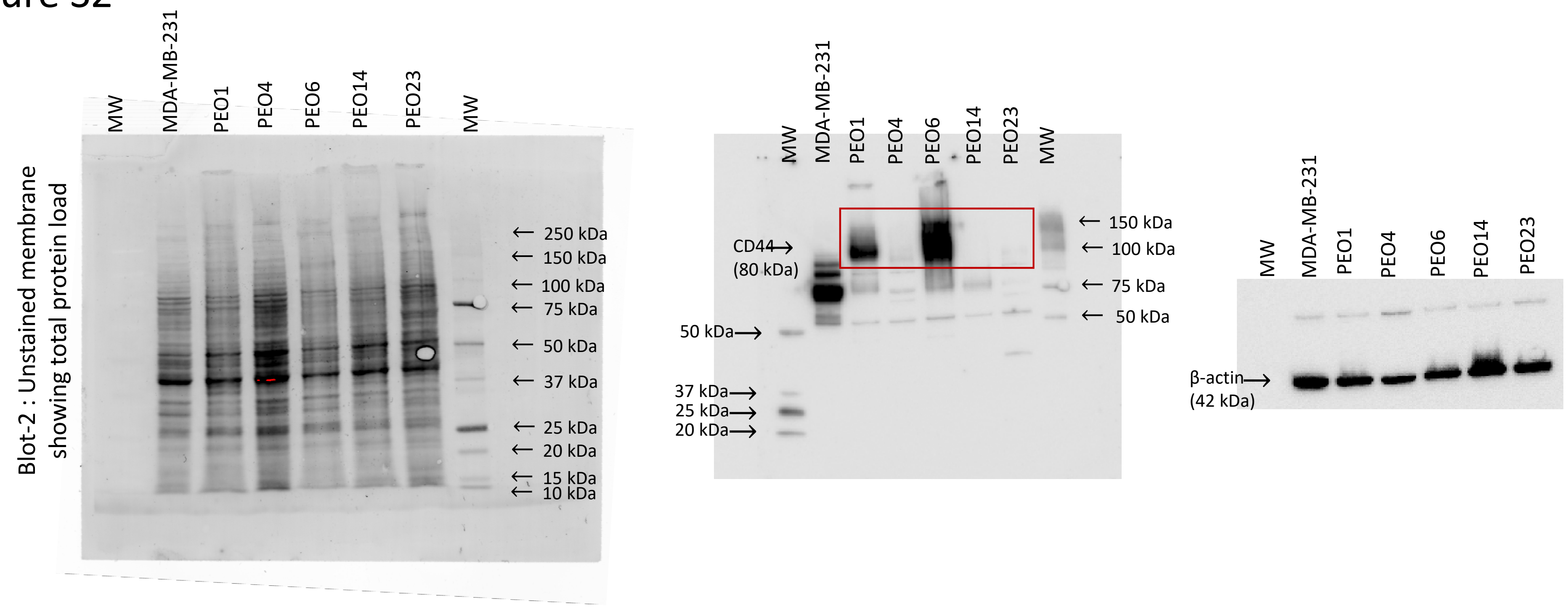

Proteins collected from MDA-MB-231 and HGSOC cells were resolved on a 10% gel (TGX stain free fast cast acrylamide) via electrophoresis (200 V). The gel was then activated using UV light and a Trans-Blot® Turbo™ Transfer System was used to transfer the resolved proteins to a PVDF membrane. After transfer, total protein load was visualized on the unstained membrane using BioRad ChemiDoc imager. After 1 hour of blocking with 5% non-fat dry milk, blot 2 was was incubated for CD44 and β-actin.

Figure S2

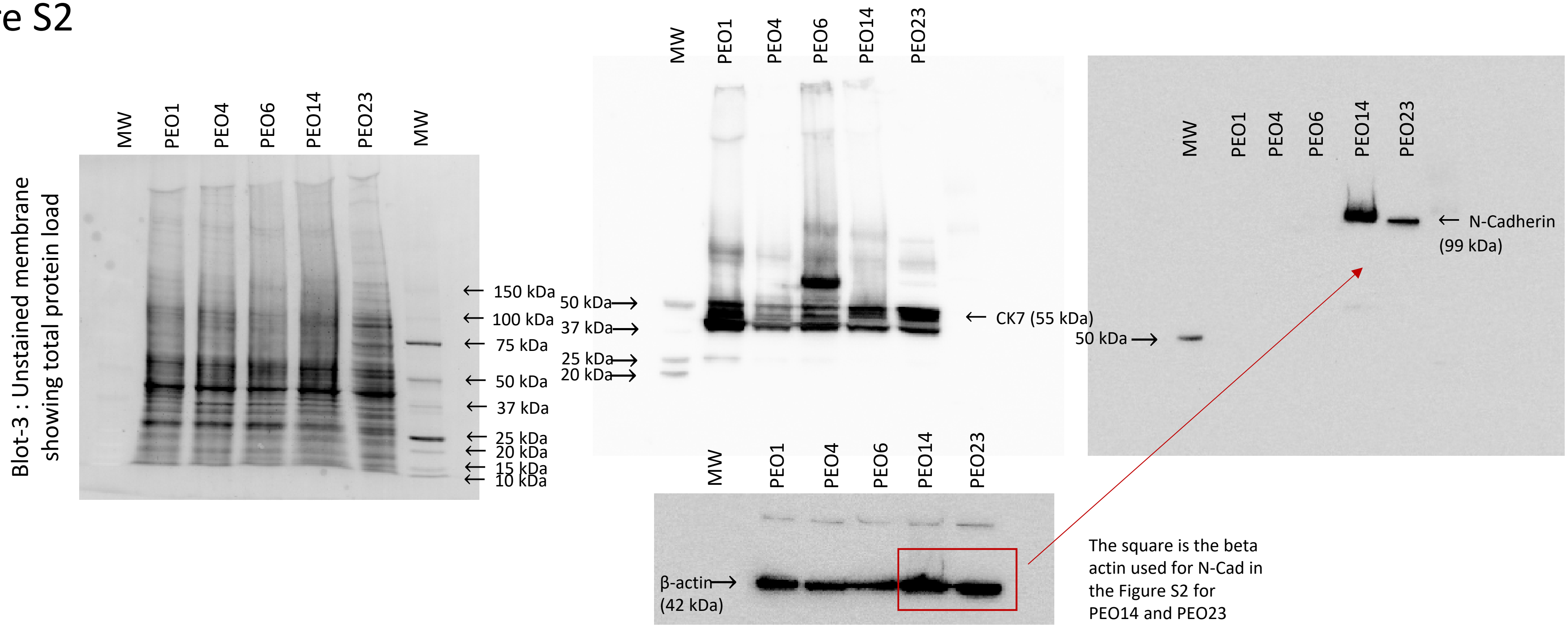

Proteins collected from HGSOC cells were resolved on a 10% gel (TGX stain free fast cast acrylamide) via electrophoresis (200 V). The gel was then activated using UV light and a Trans-Blot® Turbo™ Transfer System was used to transfer the resolved proteins to a PVDF membrane. After transfer, total protein load was visualized on the unstained membrane using BioRad ChemiDoc imager. After 1 hour of blocking with 5% non-fat dry milk, blot 3 was incubated for CK7, N-Cadherin and β-actin.

Figure S2

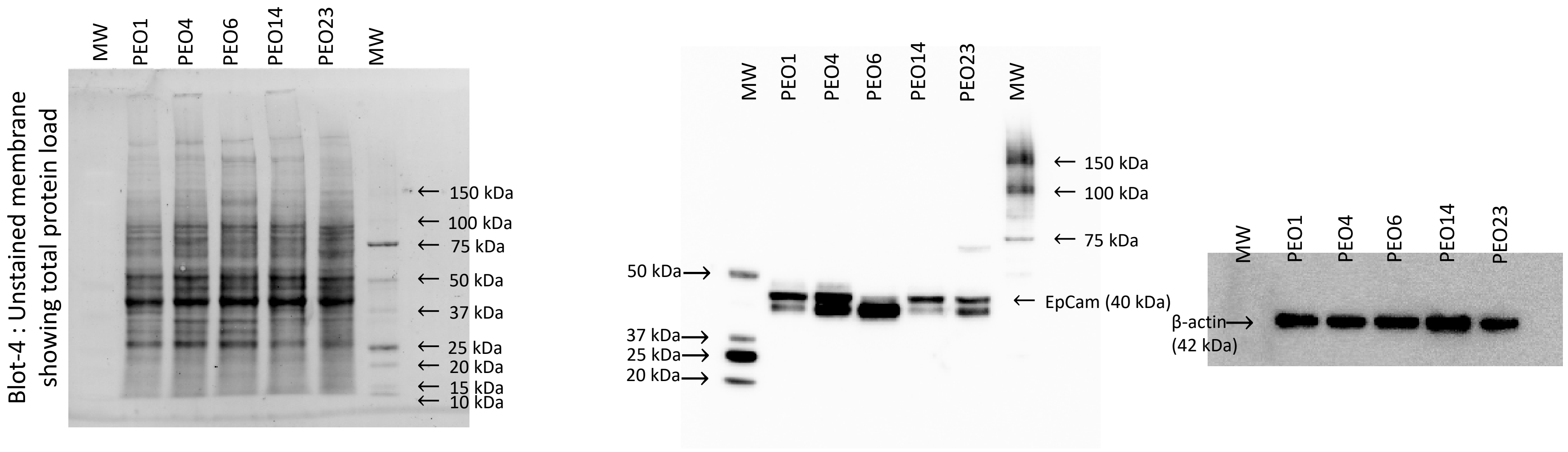

Proteins collected from HGSOC cells were resolved on a 10% gel (TGX stain free fast cast acrylamide) via electrophoresis (200 V). The gel was then activated using UV light and a Trans-Blot® Turbo™ Transfer System was used to transfer the resolved proteins to a PVDF membrane. After transfer, total protein load was visualized on the unstained membrane using BioRad ChemiDoc imager. After 1 hour of blocking with 5% non-fat dry milk, blot 4 was incubated for EpCam and β-actin.

Figure S2

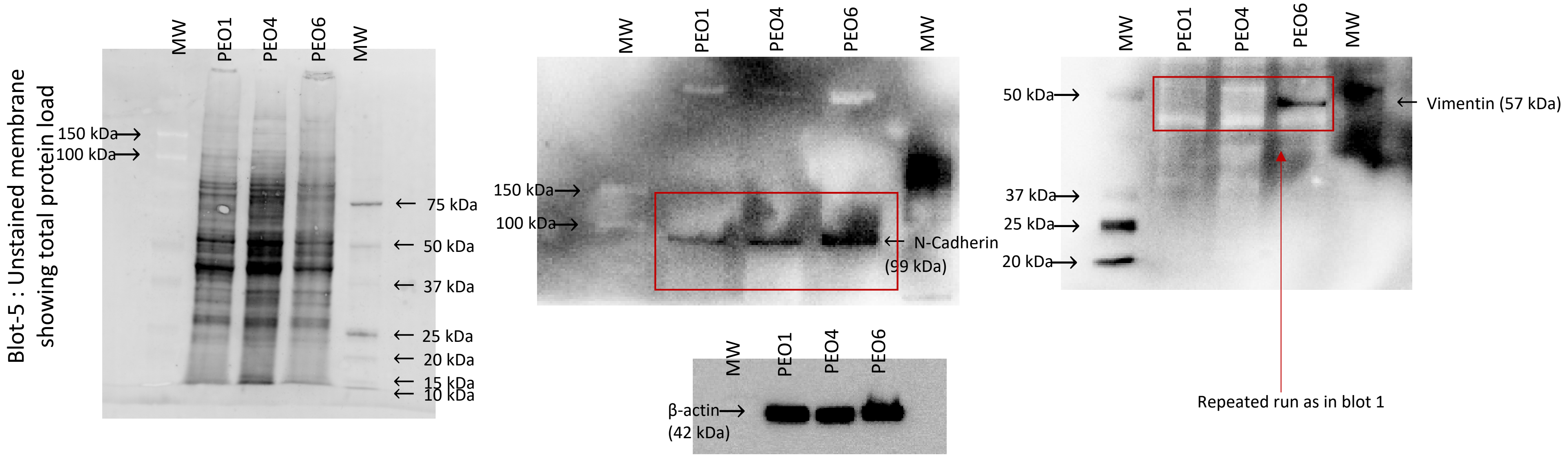

Proteins collected from HGSOC cells were resolved on a 10% gel (TGX stain free fast cast acrylamide) via electrophoresis (200 V). The gel was then activated using UV light and a Trans-Blot® Turbo™ Transfer System was used to transfer the resolved proteins to a PVDF membrane. After transfer, total protein load was visualized on the unstained membrane using BioRad ChemiDoc imager. After 1 hour of blocking with 5% non-fat dry milk, blot 1 was cut at75 kDa. The part below 75 kDa was incubated for vimentin and β-actin and the part above 75 kDa was incubated for N-Cadherin. Two stained molecular weight ladders on each side were used for cutting accuracy.

Figure S7

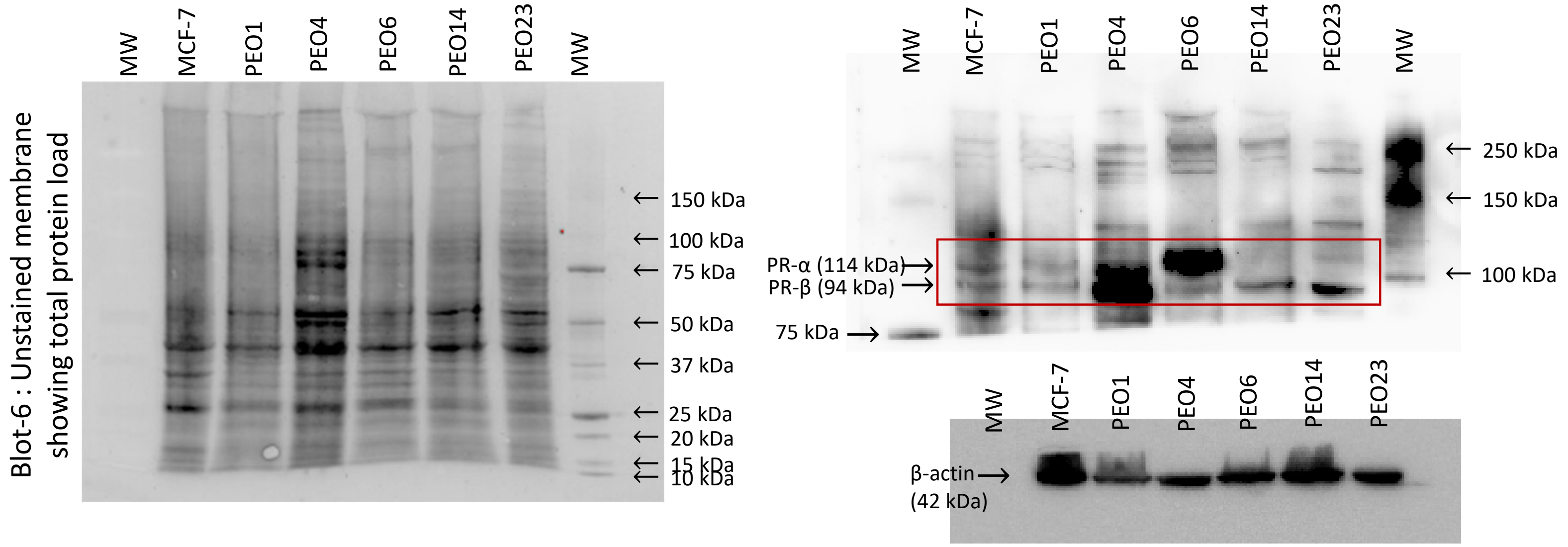

Proteins collected from MCF-7 and HGSOC cells were resolved on a 10% gel (TGX stain free fast cast acrylamide) via electrophoresis (200 V). The gel was then activated using UV light and a Trans-Blot® Turbo™ Transfer System was used to transfer the resolved proteins to a PVDF membrane. After transfer, total protein load was visualized on the unstained membrane using BioRad ChemiDoc imager. After 1 hour of blocking with 5% non-fat dry milk, blot 6 was cut at 75 kDa. The part below 75 kDa was incubated for β-actin and the part above 75 kDa was incubated for PR. Two stained molecular weight ladders on each side were used for cutting accuracy.

Figure S7

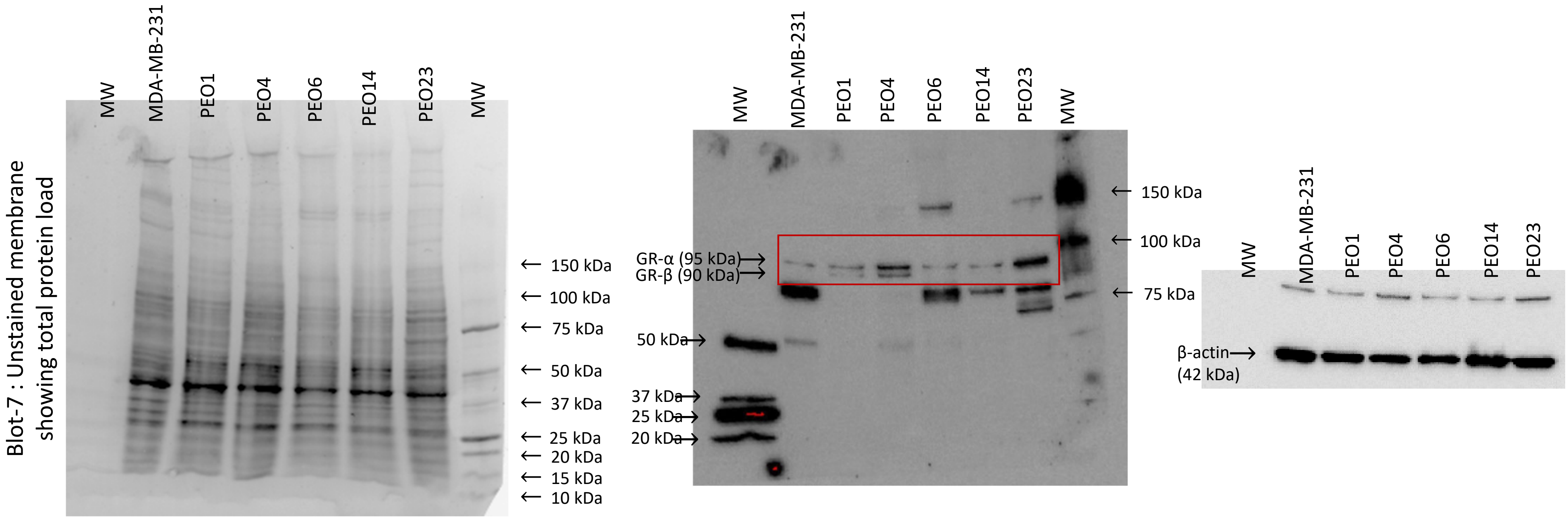

Proteins collected from MDA-MB-231 and HGSOC cells were resolved on a 10% gel (TGX stain free fast cast acrylamide) via electrophoresis (200 V). The gel was then activated using UV light and a Trans-Blot® Turbo™ Transfer System was used to transfer the resolved proteins to a PVDF membrane. After transfer, total protein load was visualized on the unstained membrane using BioRad ChemiDoc imager. After 1 hour of blocking with 5% non-fat dry milk, blot 7 was incubated for GR and β-actin.
